# Antioxidant and Neuroprotective Effects of Seed Oils from *Trichosanthes kirilowii* and *T. laceribractea* in *Caenorhabditis elegans*: A Comparative Analysis and Mechanism Study

**DOI:** 10.3390/antiox13070861

**Published:** 2024-07-18

**Authors:** Wenqian Wang, Shan Li, Yunguo Zhu, Xianghuan Cui, Zhejin Sheng, Hongbing Wang, Zhou Cheng

**Affiliations:** School of Life Sciences and Technology, Tongji University, Shanghai 200092, China; 2110808@tongji.edu.cn (W.W.); lishanbio@tongji.edu.cn (S.L.); ygzhu@tongji.edu.cn (Y.Z.); cuixh@mail.tongji.edu.cn (X.C.); zhejinsheng@tongji.edu.cn (Z.S.); hbwang@tongji.edu.cn (H.W.)

**Keywords:** *T. kirilowii* Maxim, *T. laceribractea* Hayata, seed oil, antioxidant activity, neuroprotective activity

## Abstract

Excess reactive oxygen species (ROS) can accelerate amyloid β (Aβ) aggregation and tau protein hyperphosphorylation in neuron cells, which further leads to neurodegenerative diseases such as Alzheimer’s disease (AD). Therefore, there is an urgent need to find natural and safe antioxidants for preventing or treating such neurodegenerative diseases. The seeds of *Trichosanthes kirilowii* Maxim and *T. laceribractea* Hayata have long been used for medicinal and edible purposes in China. However, the antioxidant and neuroprotective activities and underlying mechanisms of their seed oils still remain unclear. Herein, we examine the antioxidant and neuroprotective effects of seed oils extracted from different germplasms, *T. kirilowii* (YNHH and SDJN) and *T. laceribractea* (ZJQT and SXHZ), on ROS levels and neuroprotective activities in *C. elegans*. The results demonstrated that the seed oils significantly reduced the ROS levels in *C. elegans* by 17.03–42.74%, with *T. kirilowii* (YNHH and SDJN) exhibiting significantly stronger ROS scavenging abilities than *T. laceribractea* (ZJQT and SXHZ). The seed oils from *T. kirilowii* (YNHH and SDJN) alleviated the production and aggregation of Aβ and the phosphorylation and polymerization of tau, suggesting a potential neuroprotective role. Conversely, seed oils from *T. laceribractea* (ZJQT and SXHZ) show minimal neuroprotective effects in *C. elegans*. These differential outcomes might stem from distinct mechanisms underlying antioxidant and neuroprotective effects, with the *ctl-2* gene implicated as pivotal in mediating the significant neuroprotective effects of seed oils from *T. kirilowii* (YNHH and SDJN). Our findings have provided valuable insights into the antioxidant and neuroprotective properties of *T. kirilowii* seed oils, paving the way for further research aimed at elucidating the underlying mechanisms and exploring their potential therapeutic applications in combating neurodegenerative diseases.

## 1. Introduction

The *Trichosanthes* L. genus within the Cucurbitaceae family includes 84 species and eight variants with a global distribution, mainly in eastern and southern Asia and northern Australia [1]. Among these, *T. kirilowii* Maxim and *T. laceribractea* Hayata stand out as widely utilized species within the genus. The dried mature fruits, seeds, peels, and roots of *T. kirilowii* hold a prominent place in traditional Chinese medicine for their use in loosening bowels and treating cardiovascular and cerebrovascular diseases [2]. The seeds of both *T. kirilowii* and *T. laceribractea* also have long been used as snack foods following frying and other processing methods in China [3], which are notably rich in fat and protein and particularly abundant in unsaturated fatty acids (UFAs), such as trichosanic acid [4]. Despite their nutritional value, the full extent of their potential biological activities and functions remains to be elucidated. Our previous study has suggested that the seed oils from *T. kirilowii* and *T. laceribractea* could enhance oxidative stress tolerance and delay aging in *C. elegans* [5]. However, their further antioxidant and neuroprotective effects and the underlying mechanism involved warrant further investigation.

ROS are highly reactive molecules derived from molecular oxygen (O_2_), originating from both endogenous sources, such as cellular organelles and inflammatory cells, and exogenous sources, such as ionizing radiation, alcohol, food, tobacco, chemotherapeutical agents, and infectious agents [6]. The accumulated excessive ROS can induce oxidative stress, a pivotal factor in the pathogenesis of various neurodegenerative disorders, including Alzheimer’s disease (AD), Parkinson’s disease (PD), and Huntington’s disease (HD), presenting significant challenges to global healthcare systems [7]. AD, the most prevalent progressive neurologic disorder, manifests with impairments in memory, cognition, and behavior. Histopathologically, AD is characterized by the accumulation of neurofibrillary tangles composed of hyperphosphorylated tau protein and amyloid β (Aβ) plaques in the brain tissue, contributing to neuronal degeneration or death [8]. ROS are implicated in the pathogenesis of AD, as they can increase the production and aggregation of Aβ and promote the phosphorylation and polymerization of tau [9]. Endogenous enzymes and molecules, including superoxide dismutase (SOD), catalase (CAT), glutathione reductase (GR), glutathione peroxidase (GPx), and glutathione (GSH), play vital roles in detoxifying ROS [10]. However, under persistent and overwhelming oxidative stress, organisms may require externally supplied active substances to maintain homeostasis [11]. Hence, the discovery of natural antioxidants and neuroprotective agents is crucial for alleviating symptoms and delaying the progression of AD. In recent years, there has been a notable surge in the utilization of plant-derived medicines for disease prevention and treatment [12].

*C. elegans* stands out as a multicellular model organism endowed with numerous advantages, including its diminutive size, low maintenance costs, ease of culturing, short generation cycles, and straightforward genetic manipulation. Importantly, *C. elegans* harbors a plethora of genes and pathways homologous to those found in humans [13]. Furthermore, its fully mapped neuronal network renders it exceptionally suited for neurobiological research [14]. Transgenic *C. elegans* strains expressing human Aβ and tau have been instrumental in establishing AD model systems for screening potential therapeutic agents and unraveling their molecular mechanisms [15]. Our previous observations underscored the variability in antioxidant and anti-aging activities of seed oils between species, with *T. kirilowii* demonstrating more potent effects in *C. elegans* compared to *T. laceribractea* [5]. Therefore, we speculated that the seed oils from *T. kirilowii* and *T. laceribractea* are likely to have different ROS scavenging ability and neuroprotective activities, along with the underlying mechanisms implicated in mitigating neurodegenerative diseases. In this study, two germplasms of *T. kirilowii* and two germplasms of *T. laceribractea* were selected, each exhibiting distinct antioxidant activities and fatty-acid compositions [5], for comprehensive activity detection and mechanism exploration. Initially, we assessed the ROS scavenging ability of seed oils from *T. kirilowii* and *T. laceribractea* in wild-type *C. elegans* N2. Subsequently, we evaluated their neuroprotective activity using various assays, including paralysis, thrashing, chemotaxis, and sensitivity to 5-Hydroxytryptamine (5-HT), in several AD model *C. elegans* strains, such as CL4176, CL2122, and CL2355 (Aβ-induced neuronal damage); VH254 (tau-induced neuronal damage); and PHX3692 (damage in GABA neurons). Finally, we performed RNA sequencing (RNA-seq) and analyzed the activity of antioxidant enzymes and levels of antioxidant molecules to decipher the related mechanisms of action influenced by the seed oils from *T. kirilowii* and *T. laceribractea*. These findings promise to enrich our comprehension of the disparities in antioxidant and neuroprotective activities, as well as the associated mechanisms of action, between the seed oils of *T. kirilowii* and *T. laceribractea*.

## 2. Materials and Methods

### 2.1. Materials and Seed Oil Preparation

Two germplasms, YNHH (Honghe, Yunnan Province, 103°22′32.16″ E, 23°21′51.19″ N) and SDJN (Jining, Shandong Province, 116°35′47.36″ E, 35°24′29.52″ N), of *T. kirilowii* Maxim and two germplasms, ZJQT (Qingtian, Zhejiang Province, 120°17′22.38″ E, 28°08′28.53″ N) and SXHZ (Hanzhong, Shanxi Province, 107°01′54.98″ E, 33°04′4.22″ N), of *T. laceribractea* Hayata were selected based on the distinct antioxidant activities and fatty-acid compositions of their seed oils, which contained five major fatty acids: linoleic acid (LA, 33.08–39.46%), trichosanic acid (TA, 25.54–45.53%), oleic acid (OA, 6.98–29.30%), palmitic acid (PA, 4.17–7.25%), and stearic acid (SA, 2.60–3.45%), as demonstrated in our previous study [5]. Their seed oils were extracted using the Soxhlet extraction method, following established protocols [16]. Linseed oil (MedChemExpress, Monmouth Junction, NJ, USA) was utilized as a positive control group for comparative purposes.

### 2.2. C. elegans Strains and Maintenance Conditions

The *C. elegans* strains were acquired from the Caenorhabditis Genetics Center (CGC; Minneapolis, MN, USA). These strains included the Bristol wild-type N2, as well as transgenic strains: CL4176 [(pAF29) myo-3p:Aβ1-42+(pRF4) rol-6 (su1006)], VH254 [F25B3.3:tau352(PHP)+pha-1(+)], CL2355 [pCL45 (snb-1:Abeta1-42:30-UTR (long)+mtl-2:GFP], and CL2122 [(pPD30.38) unc-54 (vector)+(pCL26) mtl-2:GFP]. The PHX3692 [unc-47p::mcherry+rol-6] strain was constructed by our laboratory. The *C. elegans* strains were cultured at 20 °C (unless otherwise specified) on solid nematode growth medium (NGM) plates or in 96-well plates containing Escherichia coli OP50 as the food source [17]. To prevent progeny hatching, 5-fluorodeoxyuridine (FUDR) was added to the culture medium.

### 2.3. Measurement of Reactive Oxygen Species (ROS)

Evaluated levels of ROS can induce oxidative stress, culminating in cellular injury and neuronal cell demise [18]. To quantify endogenous ROS levels, we employed 2,7-dichlorofluorescein diacetate (H2DCF-DA) (Sigma-Aldrich, St. Louis, MO, USA), a compound that reacts with endogenous ROS to generate a fluorescent product. Synchronized L4-stage worms of the N2 strain were transferred onto NGM plates treated with 200 μg/mL of seed oil for a duration of 3 d. Subsequently, the worms were carefully washed and transferred into new 96-well plates, with each well containing 15 worms, and exposed to 50 µM H_2_DCF-DA for 1 h at 37 °C. Fluorescence intensity was then measured using excitation and emission wavelengths of 485 nm and 535 nm, respectively. Linseed oil was utilized as the positive control, while untreated worms served as the control group. This assay was repeated five times to ensure accuracy and reproducibility of the results.

### 2.4. Paralysis Assay

Extracellular deposition of Aβ is a hallmark of AD pathogenesis, exerting neurotoxic and myotoxic effects [19]. The *C. elegans* strain CL4176, harboring a temperature-sensitive mutation, expresses human Aβ_1–42_. This aggregates within muscle cells, leading to paralysis in the worms [20]. For the paralysis assay, synchronized L1-stage worms were transferred onto NGM plates treated with 200 μg/mL of seed oil and cultured at 16 °C for 36 h. Subsequently, the temperature was increased to 23 °C to induce transgenic expression, and paralyzed worms were scored hourly over a 28 h period at 23 °C. Linseed oil was utilized as the positive control, while untreated worms served as the control group. Worms were considered paralyzed if they exhibited a lack of movement and failed to respond to stimulus from a platinum wire or displayed an anterior halo. The assay was conducted in triplicate, and the PT_50_ (time duration in which half of the worms were paralyzed) was calculated for each treatment condition.

### 2.5. Thrashing Assay

The VH254 stain of *C. elegans* is characterized by hyperphosphorylation of tau protein, leading to impaired locomotion [21]. For the thrashing assay, synchronized L1-stage worms were transferred into 96-well plates, with 30 worms per well, containing 200 μg/mL of seed oil. The plates were then incubated at 20 °C for 3 d. Subsequently, thrashing rates were assessed under an optical microscope. A single thrash was defined as a complete change in the direction of the body along the midline, and the number of body thrashes in 10 s was recorded. Linseed oil was employed as the positive control, with untreated worms serving as the control group. The assay was performed in triplicate to ensure robustness and reproducibility of the results.

### 2.6. Chemotaxis Assay

The chemotaxis response in *C. elegans* involves the activation of sensory neurons and interneurons, ultimately stimulating motor neurons [22]. In the transgenic *C. elegans* strain CL2355, the synaptobrevin orthologous (*snb-1*) promoter drives pan-neuronal expression of Aβ, resulting in defects in chemotaxis to attractants and 5-Hydroxytryptamine (5-HT) sensitivity. The *C. elegans* strain CL2122 had the same genetic background as CL2355 except for not expressing the Aβ protein [23]. For the chemotaxis assay, synchronized L1-stage worms of the transgenic strain CL2355 and its control strain CL2122 were treated with 200 μg/mL of seed oil and cultured at 16 °C for 36 h before being increased to 23 °C for an additional 36 h. Equal volumes of 1 M sodium acetate and 1 M sodium azide were blended to create an attractant solution, while a control odorant was prepared using a mixture of 1 M sodium azide and sterile water. The worms were then washed and placed in the center of a clear 10 cm NGM plate, with 10 μL of attractant dropped onto one side and 10 μL of control odorant dropped onto the other side. After 1 h, the number of worms in the attractant quadrants (A_1_) and control quadrants (A_2_) were recorded. The chemotaxis index (CI) was calculated using the formula:

$$\mathrm{CI}=\left(A_{1}-A_{2}\right)/\left(\mathrm{total}\;\mathrm{number}\;\mathrm{of}\;\mathrm{scored}\;\mathrm{worms}\right)$$

Linseed oil was employed as the positive control, while untreated worms served as the control group. The assay was conducted in triplicate to ensure reliability and consistency of results.

### 2.7. 5-HT Sensitivity Assay

5-HT is a pivotal neurotransmitter that regulates various behaviors in *C. elegans* [24]. In the CL2355 strain, where Aβ is expressed in neuronal cells, exposure to exogenous 5-HT induces paralysis. For the 5-HT sensitivity assay, synchronized L1-stage worms of the transgenic strain CL2355 and its control strain CL2122 were treated with 200 μg/mL of seed oil and cultured at 16 °C for 36 h before being increased to 23 °C for an additional 36 h. Following incubation, the worms were collected using M9 buffer, and the number of paralyzed worms after exposure to 5 mg/mL of 5-HT in a 96-well plate for 24 h was counted. Linseed oil served as the positive control, while untreated worms served as the control group. The assay was conducted in triplicate to ensure robustness and reproducibility of the results.

### 2.8. Chemotaxis Assay

γ-aminobutyric acid (GABA) is a crucial amino acid neurotransmitter involved in the regulation of motor functions in *C. elegans* [25]. In the transgenic *C. elegans* strain PHX3692, GABAergic neurons are visualized through translational expression of mCherry driven by the promoter of the γ-aminobutyric acid transporter (unc-47p::mCherry). For the GABA neuron assay, synchronized L4-stage worms were transferred onto plates containing 200 μg/mL of seed oil and cultured for 3 d. Subsequently, the worms were carefully washed and mounted on 2% agar pads to enable visualization under a fluorescence microscope (Olympus BX53, Tokyo, Japan). The images obtained were used to count the number of worms exhibiting two or more instances of loss of neuronal soma or neurite in GABAergic neurons. Linseed oil served as the positive control, while untreated worms served as the control group. Each experimental group consisted of at least 30 worms, and the assay was conducted in triplicate to ensure accuracy and reliability of the results.

### 2.9. RNA Sequencing

Synchronized L4-stage wild-type worms were cultured on NGM plates treated with the four seed oils from *T. kirilowii* (YNHH and SDJN) and *T. laceribractea* (ZJQT and SXHZ) for 3 d, with untreated worms serving as the control. Subsequently, the worms were washed with M9 buffer three times, and total RNA was isolated using TRIzol (Invitrogen, Waltham, MA, USA) following the manufacturer’s instructions. Gene expression analysis was conducted by Novo Gene Corporation (Beijing, China). RNA sequencing (RNA-seq) was performed on each experimental group, with three independent biological replicates for each condition. Differential expression analysis was conducted to identify genes that were differentially expressed between the experimental and control groups of worms. These differentially expressed genes (DEGs) were further annotated using Gene Ontology (GO) terms. Additionally, further classification was carried out using the Kyoto Encyclopedia of Genes and Genomes (KEGG) pathway database. Subsequently, genes involved in the synthesis of antioxidant enzymes and molecules, such as superoxide dismutase (SOD), catalase (CAT), glutathione reductase (GR), glutathione peroxidase (GPx), and glutathione (GSH), were analyzed to determine whether they exhibited differential expression between the experimental and control groups.

### 2.10. Determination of GSH Level and CAT Activity

Synchronized L4-stage wild-type worms were cultivated on NGM plates treated with the four seed oils from *T. kirilowii* (YNHH and SDJN) and *T. laceribractea* (ZJQT and SXHZ) for 3 d, with untreated worms serving as the control. Subsequently, the worms were washed with M9 buffer three times. The levels of GSH and CAT activity in *C. elegans* were determined using assay kits (Shanghai Enzyme-linked Biotechnology Co., Ltd., Shanghai, China), following the instructions provided. The GSH can react with DNTB (5,5’-dithiobis (2-nitrobenzoic acid)) to produce yellow TNB (5-nitro-2-mercaptobenzoic acid), which has an absorption at 412 nm. The content of GSH could be measured by determining its absorbance at 412 nm. The CAT could decompose hydrogen peroxide (H_2_O_2_), which has an absorption at 240 nm. The activity of CAT was determined by measuring the change rate of the absorbance at 240 nm. Each assay was conducted in triplicate to ensure accuracy and reproducibility of the results.

### 2.11. Statistical Analysis

All values presented are the average of at least three biological replicates and are expressed as means ± standard deviation (SD). Graphs were generated using GraphPad Prism 8.0 software. Statistical analysis was conducted using SPSS 19.0 software. One-way analysis of variance (ANOVA) was performed to compare between groups, followed by appropriate post hoc tests for pairwise comparisons if significant differences were observed. A *p*-value less than 0.05 was considered statistically significant.

## 3. Results

### 3.1. Seed Oils Reduced ROS Levels

**Figure 1:**
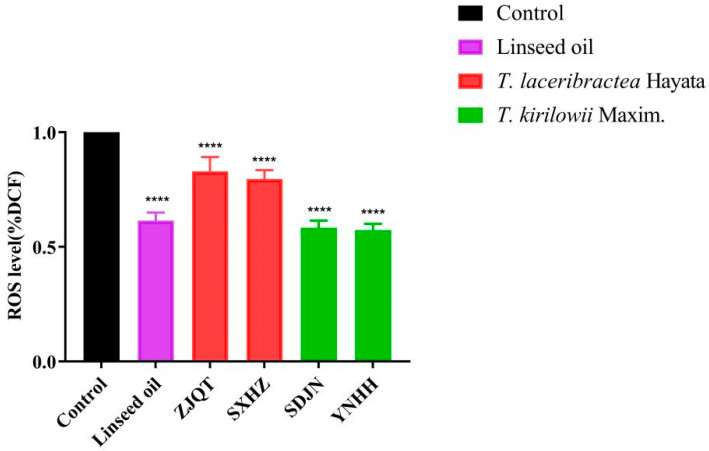
Effects of seed oils from *T. kirilowii* and *T. laceribractea* on reactive oxygen species (ROS) levels in wild-type *C. elegans* N2. **** *p* < 0.0001 vs. control. Red: germplasm ZJQT and SXHZ of *T. laceribractea*; green: germplasm SDJN and YNHH of *T. kirilowii*.

The seed oils extracted from *T. kirilowii* (YNHH and SDJN) and *T. laceribractea* (ZJQT and SXHZ) exhibited a significant reduction in reactive oxygen species (ROS) levels in wild-type *C. elegans* N2 (Figure 1). The reduction in ROS levels varied among the different seed oils, with the order of decreasing ROS percentage as follows: YNHH (42.74%), SDJN (41.68%), linseed oil (38.52%), SXHZ (20.31%), and ZJQT (17.03%) (Table S1). All four germplasms of *T. kirilowii* and *T. laceribractea* demonstrated antioxidant activity in *C. elegans*. Furthermore, the ROS scavenging activity of seed oils from *T. kirilowii* was significantly stronger compared to those from *T. laceribractea*, with activity levels similar to that of linseed oil (Figure 1, Table S1).

### 3.2. Seed Oils Delayed Aβ-Induced Paralysis

**Figure 2:**
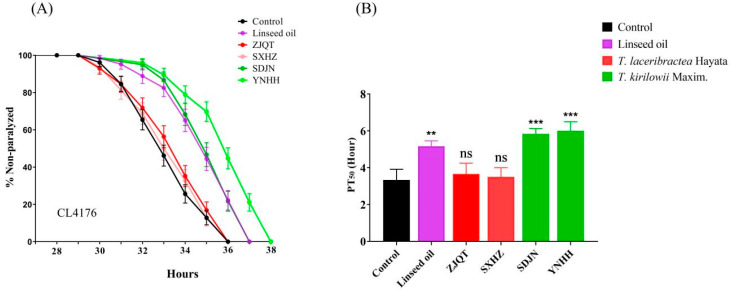
Effect of seed oils from *T. kirilowii* and *T. laceribractea* on Aβ-induced paralysis in CL4176 strain. (**A**) The paralysis curve of the CL4176 strain treated with different oils. (**B**) The PT_50_ value of the CL4176 strain treated with different oils. ** *p* < 0.01, *** *p* < 0.001, ns: no significance vs. control. Red: germplasm ZJQT and SXHZ of *T. laceribractea*; green: germplasm YNHH and SDJN of *T. kirilowii*.

The seed oils extracted from *T. kirilowii* (YNHH and ZJQT) significantly delayed the onset of paralysis in the CL4176 strain, exhibiting higher activity compared to linseed oil. In contrast, the seed oils from *T. laceribractea* (ZJQT and SXHZ) showed no significant activity (Figure 2, Table S2). The PT_50_ value for worms treated with 200 μg/mL of linseed oil and untreated control worms were 5.17 ± 0.24 h and 3.33 ± 0.47 h, respectively. Conversely, for YNHH and SXHZ, the PT_50_ values were 6.00 ± 0.41 h and 3.50 ± 0.41 h, respectively (Table S2). These results indicate a significant difference in the paralysis-delaying effect between the seed oils from *T. kirilowii* and *T. laceribractea*, with the seed oils from *T. kirilowii* demonstrating a significant delay in Aβ-induced paralysis in *C. elegans*.

### 3.3. Seed Oils Alleviated Tau-Induced Toxicity in Locomotion

**Figure 3:**
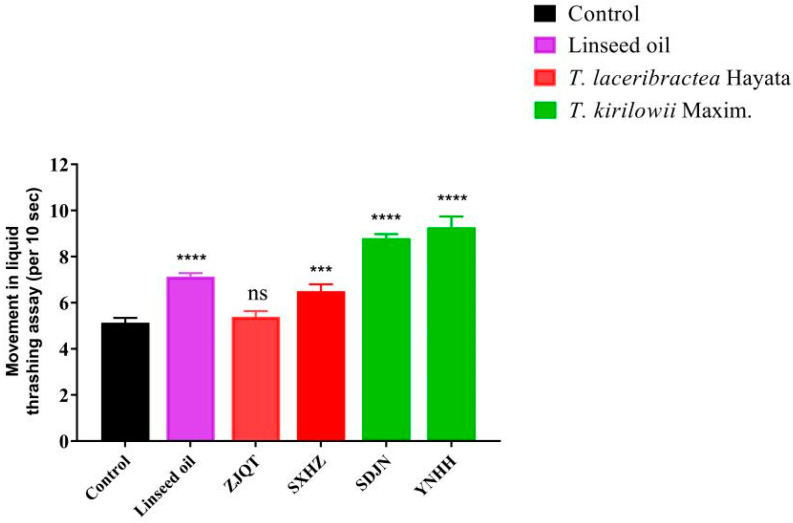
Effect of seed oils from *T. kirilowii* and *T. laceribractea* on tau-induced toxicity in locomotion in the VH254 strain. *** *p* < 0.001; **** *p* < 0.0001; ns: no significance vs. control. Red: germplasm ZJQT and SXHZ of *T. laceribractea*; green: germplasm SDJN and YNHH of *T. kirilowii*.

In the thrashing assay using the VH254 strain, worms treated with seed oils from *T. kirilowii* (YNHH and SDJN) exhibited a significant increase in thrashing rates compared to other treated groups, including those treated with linseed oil and seed oils from *T. laceribractea* (ZJQT and SXHZ) (Figure 3, Table S3). The order of thrashing rates observed in worms over 10 s was as follows: YNHH (9.27 ± 0.39), SDJN (8.80 ± 0.14), linseed oil (7.13 ± 0.12), SXHZ (6.50 ± 0.24), ZJQT (5.40 ± 0.20), and control (5.13 ± 0.17) (Table S3). While the seed oil from germplasm SXHZ of *T. laceribractea* significantly increased the trashing rate of worms compared to the control, the thrashing rate was significantly lower than that of linseed oil. Conversely, the seed oil from germplasm ZJQT of *T. laceribractea* did not significantly increase the thrashing rate of worms (Figure 3, Table S3). Therefore, the seed oils from *T. kirilowii* significantly reduced tau-induced toxicity in locomotion in *C. elegans*.

### 3.4. Seed Oils Suppressed Neuronal Aβ-Expression-Induced Defects in Chemotaxis Behavior and 5-HT Sensitivity

**Figure 4:**
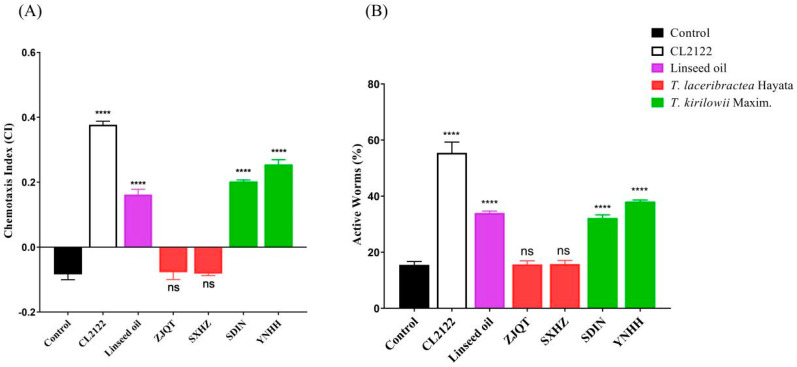
Effect of seed oils from *T. kirilowii* and *T. laceribractea* on the chemotaxis index (CI) and 5-HT sensitivity in the CL2355 strain. (**A**) Chemotaxis index. (**B**) 5-HT sensitivity. The CL2122 strain had the same genetic background as the CL2355 strain except for not expressing the Aβ protein. **** *p* < 0.0001; ns: no significance vs. control. Red: germplasm ZJQT and SXHZ of *T. laceribractea*; green: germplasm SDJN and YNHH of *T. kirilowii*.

The effect of seed oils on Aβ-induced cognitive decline was assessed in the CL2355 strain. Seed oils from *T. kirilowii* (YNHH and SDJN) significantly improved the chemotaxis index (CI) of worms, indicating enhanced sensitivity of CL2355 worms to attractants and protection of neurons against Aβ-induced damage (Figure 4A, Table S4). The highest CI observed in worms treated with seed oil from germplasm YNHH was 0.255 ± 0.012, which was significantly higher than that of germplasm SDJN (0.202 ± 0.005) from the same species of *T. kirilowii*. In comparison, the CI of CL2122 worms was 0.377 ± 0.009 (Table S4 and Figure 4A). These results suggested that the seed oils from *T. kirilowii* partially recovered the cognitive deficits of CL2355 worms. Conversely, seed oils from *T. laceribractea* (ZJQT and SXHZ) exhibited no significant activity compared to the control, and the chemotaxis indexes were significantly lower than that of linseed oil (0.162 ± 0.013) (Table S4, Figure 4A). Therefore, only the seed oils from *T. kirilowii* possessed significant neuroprotective effects in *C. elegans*.

Similarly, seed oils from *T. kirilowii* (YNHH and SDJN) also significantly improved the 5-HT sensitivity of the CL2355 worms, with a high percentage of active worms, while seed oils from *T. laceribractea* Hayata (ZJQT and SXHZ) exhibited no significant effect (Figure 4B, Table S5). The order of the percentage of active worms was as follows: CL2122 strain (55.45 ± 3.13), YNHH (38.05 ± 0.50), SDJN (32.17 ± 0.94), linseed oil (34.06 ± 0.52), SXHZ (15.82 ± 1.00), ZJQT (15.69 ± 1.02), and control (15.50 ± 0.92) (Table S5). Therefore, the seed oils of *T. kirilowii* could ameliorate phenotypic defects in *C. elegans*.

### 3.5. Seed Oils Attenuated Aβ-Induced Damage in GABA Neurons

**Figure 5:**
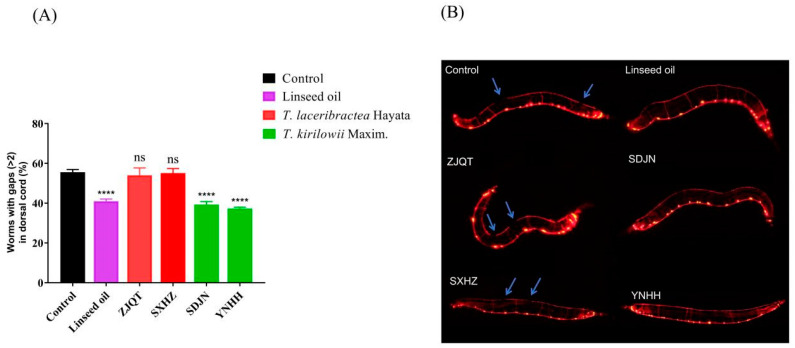
Effect of seed oils from *T. kirilowii* and *T. laceribractea* on Aβ-induced damage in GABA neurons in the PHX3692 strain. (**A**) Proportion of worms with severe neuronal damage. (**B**) Neuronal damage. The arrows indicate gaps in the dorsal cord. **** *p* < 0.0001; ns: no significance vs. control. Red: germplasm ZJQT and SXHZ of *T. laceribractea*; green: germplasm SDJN and YNHH of *T. kirilowii*.

In the transgenic *C. elegans* strain PHX3692, GABAergic neurons were labeled with red fluorescent protein, allowing for the identification of obvious damage to GABAergic neurons indicated by arrow points (Figure 5B). The percentage of worms with gaps (>2) in the dorsal cord was observed in the following order: YNHH (37.26 ± 0.59), SDJN (39.30 ± 1.18), linseed oil (41.00 ± 0.86), ZJQT (54.05 ± 2.99), SXHZ (55.11 ± 1.87), and control (55.56 ± 1.13) (Table S6). Treatment with seed oils from *T. kirilowii* (YNHH and SDJN) resulted in a significant reduction in the percentage of GABA neurons damage by 32.94% and 29.27% compared to the control, which was slightly higher than the 26.20% reduction observed in worms treated with linseed oil (Figure 5A, Table S6). Conversely, seed oils from *T. laceribractea* (ZJQT and SXHZ) had no significant effect, with only a 2.73% and 0.81% reduction in the percentage of GABA, respectively (Table S6). Therefore, the seed oils from *T. kirilowii* exhibited neuroprotective potential and helped maintain neuronal integrity in *C. elegans*.

### 3.6. Seed Oils from Different Trichosanthes Germplasms Exerted Neuroprotective Effect Based on Different Mechanisms

**Figure 6:**
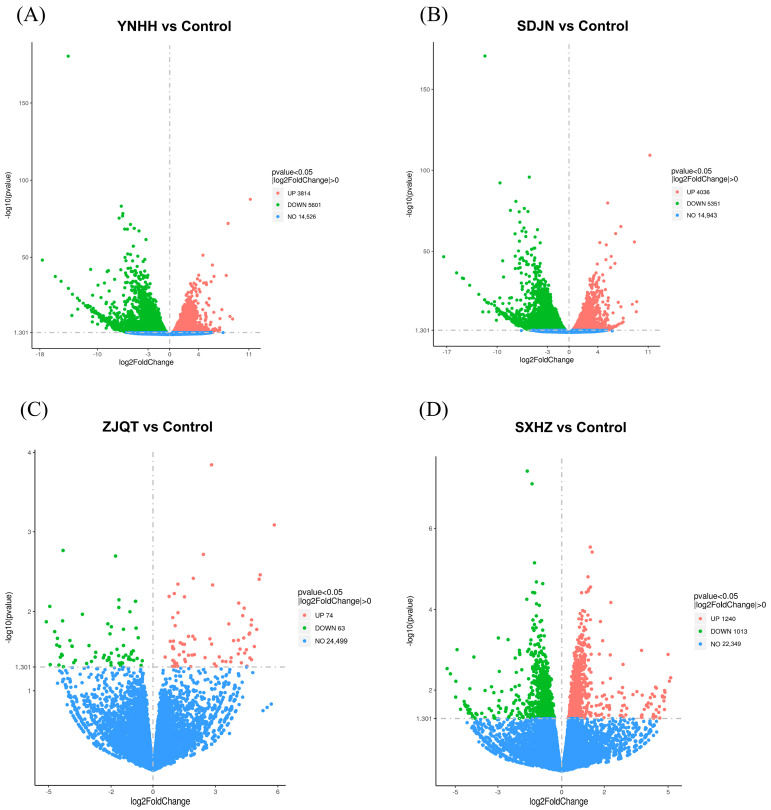
Volcano plot of differential expression genes (DEGs) in *C. elegans* N2 treated with seed oils from *T. kirilowii* (YNHH and SDJN) and *T. laceribractea* (ZJQT and SXHZ). (**A**) Control vs. YNHH. (**B**) Control vs. SDJN. (**C**) Control vs. ZJQT. (**D**) Control vs. SXHZ. Each point represents a gene; the abscissa represents the log_2_ value of the difference multiple; the ordinate represents the negative logarithm of the *p* value. Red: up-regulated; green: down-regulated; blue: genes that are not differentially expressed.

The RNA-seq analysis revealed significant differences in the number of differential expression genes (DEGs) between untreated worms (control) and worms treated with seed oils from *T. kirilowii* (YNHH and SDJN) and *T. laceribractea* (ZJQT and SXHZ), respectively (Table 1). More than 7000 total DEGs were detected in worms treated with seed oils from *T. kirilowii* (YNHH and SDJN), whereas only hundreds of total DEGs were affected by seed oils from *T. laceribractea* (ZJQT and SXHZ) (Table 1). This indicates that seed oils from *T. kirilowii* might significantly influence the physiological state of *C. elegans* by up-regulating or down-regulating the expressions of a large number of genes. Additionally, differences in the number of DEGs were observed between worms treated with seed oils from different germplasms of *T. kirilowii* or *T. laceribractea* (Table 1), which indicated there might be distinct mechanisms between seed oils from different germplasms within species. The volcano plots illustrated the distinct expression patterns of DEGs between control vs. *T. kirilowii* (YNHH and SDJN) (Figure 6A,B) and control vs. *T. laceribractea* (ZJQT and SXHZ) (Figure 6C,D). Furthermore, the differential expressions of control vs. *T. kirilowii* (YNHH and SDJN) exhibited similar patterns (Figure 6A,B), whereas those of *T. laceribractea* (ZJQT and SXHZ) showed different patterns (Figure 6C,D). These results suggest that the roles and mechanisms of seed oils may vary not only between but also within species of *Trichosanthes* L.

**Figure 7:**
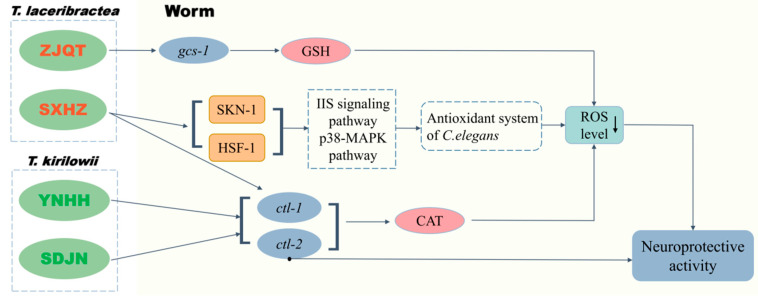
The proposed mechanism of antioxidant and neuroprotective effect exerted by different seed oils from *T. kirilowii* (YNHH and SDJN) and *T. laceribractea* (ZJQT and SXHZ) in *C. elegans*. *gcs-1*: glutathione (GSH) synthesisgene; *ctl-1*, *clt-2*: catalase (CAT) synthesis genes; *hsf-1*, *skn-1*: synthesis genes of transcription factors HSF-1 and SKN-1.

The Gene Ontology analysis revealed distinct enriched GO terms among the groups (Figure S1). Major alterations in biological process (BP), molecular function (MF), and cellular component (CC) were observed between the two species of *T. kirilowii* (SDJN and YNHH) and *T. laceribractea* (ZJQT and SXHZ) (Figure S1). In the *T. kirilowii* groups, alternations were primarily related to cell communication, signaling, and structural molecules, while in the *T. laceribractea* groups, they were associated with receptors, lipids, and extracellular regions. KEGG analysis also revealed differences in the potential signal pathways regulated by the DEGs among the four seed oil treatment groups (Figure S2). In the ZJQT group, the pathways mainly involved mucin type and other types of O-glycan biosynthesis. Conversely, in SXHZ group, pathways included axon regeneration, mTOR signaling, nucleocytoplasmic transport, longevity regulation, and RNA polymerase. However, the pathways were similar between the two germplasms (SDJN and YNHH) of *T. kirilowii*, with ribosome-related pathways being the focus. GO and KEGG annotation of the DEGs indicated different enriched terms and pathways, suggesting that seed oils from *T. kirilowii* and *T. laceribractea* may exert a neuroprotective effect through different mechanisms. Further analysis of genes related to the antioxidant system found that the synthesis of catalase (CAT)-related genes (*ctl-1*, *ctl-2*) was significantly up-regulated in the SDJN and YNHH groups, while only *ctl-1* was significantly up-regulated in the SXHZ group. In the ZJQT group, the expression of *gcs-1*, essential for intracellular GSH synthesis in *C. elegans*, was significantly up-regulated, suggesting enhanced GSH synthesis in treated worms. Additionally, the expression of synthesis genes of key transcription factors involved in classical pathways related to aging and oxidative stress response of *C. elegans* were significantly up-regulated in the SXHZ group, including *hsf-1* and *skn-1* (Table 2). This indicated that the antioxidant effect exerted by SXHZ were mediated by HSF-1 (involved in insulin/IGF signaling pathway) and SKN-1 (involved in p38-MAPK pathway). These results demonstrated that seed oils from *T. kirilowii* and *T. laceribractea* exerted an antioxidant effect through different mechanisms, with variations also observed between the two germplasms ZJQT and SXHZ of *T. laceribractea* (Figure 7). The differences in antioxidant mechanisms of seed oils from different species and germplasms may contribute to their variations in neuroprotective effects.

## 4. Discussion

Oxidative stress stands as one of the classical pathogenic hypotheses of neurodegenerative diseases, closely linked to the aging process and degenerative alternations in neurons [26]. Many neurodegenerative diseases, including AD, PD, and HD, have direct or indirect connections to the damage induced by oxidative stress. Targeting oxidative stress and identifying antioxidants with potential neuroprotective effects for the prevention and treatment of AD and other related diseases represent effective approaches [27]. In recent years, natural products have emerged as promising sources of antioxidants, presenting potential therapeutic avenues for neurodegenerative disorders [28]. Reactive oxygen species (ROS), although produced under normal physiological conditions, can lead to cellular injury and neuronal cell death when present in elevated levels [19]. *Camellia oleifera* seed oil could significantly reduce the content of ROS in *C. elegans* by 21.54%, and various plant seed oils, including linseed oil [29], pomegranate seed oil [30], and perilla frutescens seed oil [31], among others, have been shown to possess favorable antioxidant activity by reducing ROS levels. Our previous study demonstrated that seed oils derived from *T. kirilowii* and *T. laceribractea* significantly enhanced resistance to oxidative stress in *C. elegans* [5], potentially attributed to their ROS scavenging ability. In this study, it was further confirmed that there is a significant antioxidant effect in *C. elegans* following treatment with these seed oils, ranging from 17.03% to 42.74%. Moreover, the antioxidant effect was significantly positively correlated with all five neuroprotective activity indicators (r > 0.980, *p* < 0.05). Therefore, it may be speculated that the seed oils from *T. kirilowii* and *T. laceribractea* could possess neuroprotective activity, potentially contributing to the improvement of degenerative diseases.

The typical markers of AD histopathology are the hyperphosphorylated tau protein and β-amyloid (Aβ) plaques in brain tissue [8]. In this study, the seed oils from *T. kirilowii* (YNHH and SDJN) demonstrated the ability to delay the Aβ-induced paralysis in the muscle Aβ-expressing *C. elegans* strain CL4176, ameliorate chemotaxis behavior and 5-HT sensitivity in the neuron Aβ-expressing *C. elegans* strain CL2355, reduce tau-induced toxicity in locomotion in the tau protein hyperphosphorylation *C. elegans* strain VH254, and attenuate Aβ-induced damage in GABA neurons of the neuron Aβ-expressing *C. elegans* strain PHX3692. Therefore, it is suggested that the seed oil from *T. kirilowii* may alleviate the production and aggregation of Aβ and the phosphorylation and polymerization of tau, based on its ROS scavenging activity, thereby playing a neuroprotective role in *C. elegans*. However, the seed oils from *T. laceribractea* (ZJQT and SXHZ), despite demonstrating significant ROS scavenging activity, did not exhibit neuroprotective effects in *C. elegans*, except for the limited activity of the seed oil from the germplasm SXHZ to reduce tau-induced toxicity in locomotion in the strain VH254. Thus, it appears that seed oils with antioxidant activity do not necessarily imply statistically effective neuroprotective activity in *C. elegans*. Generally, the lower the antioxidant activity of the extract, the weaker its neuroprotective activity, even without significant effects on some indicators of neuroprotective activity in *C. elegans* [32].

In this study, RNA-seq analysis revealed the different mechanisms of antioxidant and neuroprotective effect produced by the seed oils from *T. kirilowii* (YNHH and SDJN) and *T. laceribractea* (ZJQT and SXHZ). The seed oils from *T. kirilowii* (YNHH and SDJN) significantly up-regulated the CAT-related genes (*ctl-1, ctl-2*) in *C. elegans*. CAT is a major ROS-scavenging enzyme in vivo and can destroy free radicals, thereby protecting neuron cells [33]. *ctl-1* and *ctl-2* are important CAT coding genes that have protective effects against ROS damage. The *ctl-1* gene is involved in the hydrogen peroxide catabolic process and the response to hydrogen peroxide, which is active in mitochondrion and peroxisome. The *ctl-2* gene is mainly located in the peroxisome and is involved in determining lifespan and peroxisome organization [34]. The inhibition of *ctl-1* and *ctl-2* gene expression has been found to decrease the antioxidant capacity of *C. elegans* [35]. Therefore, the antioxidant and neuroprotective effects of seed oils from *T. kirilowii* (YNHH and SDJN) were achieved by enhancing CAT in *C. elegans*. The seed oils from the germplasm SXHZ of *T. laceribractea* significantly up-regulated the expression of *ctl-1* and were mediated by transcription factors SKN-1 and HSF-1. The classical transcription factors SKN-1 and HSF-1 are closely related to oxidative stress and longevity pathway in *C. elegans* [36]. There are some general signaling pathways involved in the oxidative stress response of *C. elegans*, such as the insulin/IGF-1 signaling (IIS) pathway, p38 MAPK pathway, and TGF-β pathway [37], which are mediated by important transcription factors, such as DAF-16, SKN-1, and HSF-1. However, the seed oil from germplasm SXHZ of *T. laceribractea* could not significantly exert the neuroprotective effect in *C. elegans*. Similarly, the seed oil from the germplasm ZJQT of *T. laceribractea* up-regulated the expression of the *gcs-1* gene involved in GSH synthesis but did not result in neuroprotective effects in *C. elegans*, although GSH is an important intracellular molecule that protects cells against endogenous and exogenous oxidative stress [38]. Our study suggests that the significant neuroprotective effect of seed oils from *T. kirilowii* (YNHH and SDJN) is likely attributable to their significant enhancement of CAT activity in *C. elegans* through the up-regulation of *ctl-1* and *ctl-2* genes, as compared to *T. laceribractea* (ZJQT and SXHZ). Given that *ctl-2* is a key gene associated with aging and stress resistance [39], it can be inferred that *ctl-2* may play a more crucial role in mediating the neuroprotective effect observed with seed oils from *T. kirilowii* (YNHH and SDJN).

The observed biological activities of these seed oils are associated with their specific species. The seed oils from *T. kirilowii* (YNHH and SDJN) exhibited significant neuroprotective effects, while those from *T. laceribractea* (ZJQT and SXHZ) showed almost none, despite both effectively reducing ROS levels in *C. elegans*. Likewise, while the seed oils from *T. laceribractea* (ZJQT and SXHZ) significantly enhanced resistance to oxidative stress and extended the lifespan of *C. elegans*, they did not promote healthspan [5]. The significantly weaker antioxidant activity of the seed oils from *T. laceribractea* (ZJQT and SXHZ) compared to *T. kirilowii* (YNHH and SDJN) led to differences in related mechanisms of action, thereby affecting their neuroprotective and other biological activities in *C. elegans*. The significant neuroprotective effect of the seed oils from *T. kirilowii* (YNHH and SDJN) might be attributed to their significantly higher UFA (unsaturated fatty acid) content, which possesses various biological activities, including antioxidant activity [40], compared to *T. laceribractea* (ZJQT and SXHZ) [5]. Further study is needed to identify the key substances of seed oil from *T. kirilowii* that underlie its antioxidant and neuroprotective effects.

## 5. Conclusions

The seed oils from *T. kirilowii* and *T. laceribractea* exhibited antioxidant activity, significantly reducing ROS levels in *C. elegans* by 17.03–42.74%. However, while the seed oils from *T. kirilowii* demonstrated significant neuroprotective activity, those from *T. laceribractea* showed almost no neuroprotective effect in *C. elegans*. This disparity may be attributed to the differences in their ROS scavenging ability and may subsequently influence their neuroprotective activity through distinct mechanisms of action in *C. elegans*. Thus, the seed oil from *T. kirilowii* holds potential as an antioxidant and neuroprotective agent for the prevention or treatment of neurodegenerative diseases.

## Figures and Tables

**Table 1:** The number of DEGs between groups treated with different seed oils.

Groups	Total	Up-Regulating	Down-Regulating
SDJN vs. Control	7146	4511	2635
YNHH vs. Control	7065	4680	2385
ZJQT vs. Control	119	55	64
SXHZ vs. Control	415	247	168
**Inter-Species**	**Total**	**Up-Regulating**	**Down-Regulating**
SDJN vs. YNHH	574	297	277
ZJQT vs. SXHZ	327	193	134

Green: germplasm SDJN and YNHH of *T. kirilowii*; red: germplasm ZJQT and SXHZ of *T. laceribractea*.

**Table 2:** Partial differential gene expression in *C. elegans* N2 treated with seed oils from *T. kirilowii* (YNHH and SDJN) and *T. laceribractea* (ZJQT and SXHZ).

Groups	log_2_Foldchange (*p* Value)				
	*gcs-1*	*ctl-1*	*ctl-2*	*hsf-1*	*skn-1*
SDJN	0.3989 (0.12)	**1.2632 (0.01)**	**1.0020 (0.01)**	0.4389 (0.08)	0.1237 (0.66)
YNHH	0.2042 (0.39)	**0.5416 (0.02)**	**0.6183 (0.02)**	0.3950 (0.10)	0.2246 (0.40)
SXHZ	0.3614 (0.09)	**0.4787 (0.01)**	0.0657 (0.72)	**0.0165 (0.02)**	**0.7193 (0.01)**
ZJQT	**0.2411 (0.04)**	0.1000 (0.63)	0.0059 (0.97)	0.1332 (0.61)	0.0005 (1.00)

Green: germplasm SDJN and YNHH of *T. kirilowii*; red: germplasm ZJQT and SXHZ of *T. laceribractea*. Bold: *p* < 0.05. *gcs-1*: glutathione (GSH) synthesis gene; *ctl-1*, *clt-2*: catalase (CAT) synthesis genes; *hsf-1*, *skn-1*: synthesis genes of transcription factors HSF-1 and SKN-1.

## Data Availability

Data available on request from the authors.

## Supplementary Materials

The following supporting information can be downloaded at: https://www.mdpi.com/article/10.3390/antiox13070861/s1. Table S1: Effects of the seed oils from *T. kirilowii* and *T. laceribractea* on ROS levels in wild-type *C. elegans* N2. Table S2: Effect of seed oils from *T. kirilowii* and *T. laceribractea* on Aβ-induced paralysis in transgenic *C. elegans* CL4176. Table S3: Effect of seed oils from *T. kirilowii* and *T. laceribractea* on thrashing rates in transgenic *C. elegans* VH254. Table S4: Effect of seed oils from *T. kirilowii* and *T. laceribractea* on CI in transgenic *C. elegans* CL2355. Table S5: Effect of seed oils from *T. kirilowii* and *T. laceribractea* on 5-HT sensitivity in transgenic *C. elegans* CL2355. Table S6: Effect of seed oils from *T. kirilowii* and *T. laceribractea* on neuron damage in transgenic *C. elegans* PHX3692. Table S7: Effect of seed oils from *T. kirilowii* and *T. laceribractea* on GSH level in *C. elegans* N2. Table S8: Effect of seed oils from *T. kirilowii* and *T. laceribractea* on CAT activity in *C. elegans* N2. Figure S1: The significant enriched GO terms between groups. Figure S2: KEGG pathway enrichment of DEGs.
