# Supplementary material for: Antioxidant and Neuroprotective Effects of Seed Oils from Trichosanthes kirilowii and T. laceribractea in Caenorhabditis elegans: A Comparative Analysis and Mechanism Study
Source: Antioxidants (Basel). 2024 Jul 18;13(7):861. doi: 10.3390/antiox13070861 (PMC11273834; doi:10.3390/antiox13070861)
Supplement: Supplementary file 1 [file antioxidants-13-00861-s001.zip › Supplementary materials.pdf]

Table S1. Effects of the seed oils from *T. kirilowii* and *T. laceribractea* on ROS levels in wild-type *C. elegans* N2.

Groups	Relative fluorescence (%)	Change (%)
Control	100.00±0.00 C	/
YNHH	57.26±23.45 A	- 42.74
SDJN	58.32±2.81 A	- 41.68
Linseed oil	61.48±3.14 A	- 38.52
SXHZ	79.69±3.43 B	- 20.31
ZJQT	82.97±5.61 B	- 17.03

The data were analyzed by one way-ANOVA analysis and different uppercases indicated significant difference at level of 0.01 by Least-Significant Difference Test (LSD). Red: germplasm ZJQT and SXHZ of *T. laceribractea*; Green: germplasm YNHH and SDJN of *T. kirilowii*; Purple: linseed oil.

Table S2. Effect of seed oils from *T. kirilowii* and *T. laceribractea* on Aβ-induced paralysis in transgenic *C. elegans* CL4176.

Groups	PT_{50} (Hour)	Change (%)
YNHH	6.00±0.41 ^{A}	+80.18
SDJN	5.83±0.24 ^{A}	+75.08
Linseed oil	5.17±0.24 ^{A}	+55.26
ZJQT	3.67±0.47 ^{B}	+10.21
SXHZ	3.50±0.41 ^{B}	+5.11
Control	3.33±0.47 ^{B}	/

The data were analyzed by one way-ANOVA analysis and different uppercases indicated significant difference at level of 0.01 by Least-Significant Difference Test (LSD). Red: germplasm ZJQT and SXHZ of *T. laceribractea*; Green: germplasm SDJN and YNHH of *T. kirilowii*; Purple: linseed oil.

Table S3. Effect of seed oils from *T. kirilowii* and *T. laceribractea* on thrashing rates in transgenic *C. elegans* VH254.

Groups	Number of thrashing in 10S	Change (%)
Control	5.13±0.17 ^{D}	/
YNHH	9.27±0.39 ^{A}	+80.52
SDJN	8.80±0.14 ^{A}	+71.43
Linseed oil	7.13±0.12 ^{B}	+38.96
SXHZ	6.50±0.24 ^{C}	+26.62
ZJQT	5.40±0.20 ^{D}	+5.12

The data were analyzed by one way-ANOVA analysis and different uppercases indicated significant difference at level of 0.01 by Least-Significant Difference test (LSD). Red: *T. laceribractea*; Green: *T. kirilowii*; Purple: linseed oil.

Table S4. Effect of seed oils from *T. kirilowii* and *T. laceribractea* on CI in transgenic *C. elegans* CL2355.

Groups	Chemotaxis index (CI)
CL2122	0.377±0.009 ^{A}
YNHH	0.255±0.012 ^{B}
SDJN	0.202±0.005 ^{C}
Linseed oil	0.162±0.013 ^{D}
ZJQT	-0.077±0.018 ^{E}
SXHZ	-0.082±0.005 ^{E}
Control	-0.084±0.014 ^{E}

The data were analyzed by one way-ANOVA analysis and different uppercases indicated significant difference at level of 0.01 by Least-Significant Difference test (LSD). Red: *T. laceribractea*; Green: *T. kirilowii*; Purple: linseed oil.

Table S5. Effect of seed oils from *T. kirilowii* and *T. laceribractea* on 5-HT sensitivity in transgenic *C. elegans* CL2355.

Groups	Active worms (%)
CL2122	55.45±3.13 ^{A}
YNHH	38.05±0.50 ^{B}
SDJN	32.17±0.94 ^{C}
Linseed oil	34.06±0.52 ^{C}
SXHZ	15.82±1.00 ^{D}
ZJQT	15.69±1.02 ^{D}
Control	15.50±0.92 ^{D}

The data were analyzed by one way-ANOVA analysis and different uppercases indicated significant difference at level of 0.01 by Least-Significant Difference test (LSD). Red: *T. laceribractea*; Green: *T. kirilowii*; Purple: linseed oil.

Table S6. Effect of seed oils from *T. kirilowii* and *T. laceribractea* on neurons damage in transgenic *C. elegans* PHX3692.

Groups	Worms with gaps (>2) in dorsal cord (%)	Change (%)
Control	55.56±1.13 ^{B}	/
SXHZ	55.11±1.87 ^{B}	0.81
ZJQT	54.05±2.99 ^{B}	2.73
Linseed oil	41.00±0.86 ^{A}	26.20
SDJN	39.30±1.18 ^{A}	29.27
YNHH	37.26±0.59 ^{A}	32.94

The data were analyzed by one way-ANOVA analysis and different uppercases indicated significant difference at level of 0.01 by Least-Significant Difference test (LSD). Red: *T. laceribractea*; Green: *T. kirilowii*; Purple: linseed oil.

Table S7. Effect of seed oils from *T. kirilowii* and *T. laceribractea* on GSH level in *C. elegans* N2.

Groups	GSH level (μM/g tissue)
ZJQT	48.82±0.75 ^{A}
YNHH	43.20±1.23 ^{B}
Control	42.20±0.51 ^{B}
SXHZ	41.49±0.68 ^{B}
SDJN	40.77±0.81 ^{B}

The data were analyzed by one way-ANOVA analysis and different uppercases indicated significant difference at level of 0.01 by Least-Significant Difference test (LSD). Red: *T. laceribractea*; Green: *T. kirilowii*.

Table S8. Effect of seed oils from *T. kirilowii* and *T. laceribractea* on CAT activity in *C. elegans* N2.

Groups	CAT activity (nmol/min/mg tissue)
SDJN	11.22±0.07 ^{A}
YNHH	10.94±0.05 ^{B}
SXHZ	7.85±0.05 ^{C}
ZJQT	6.64±0.10 ^{D}
Control	6.58±0.07 ^{D}

The data were analyzed by one way-ANOVA analysis and different uppercases indicated significant difference at level of 0.01 by Least-Significant Difference test (LSD). Red: *T. laceribractea*; Green: *T. kirilowii*.
