# Supplementary figures and images for: Antioxidant and Neuroprotective Effects of Seed Oils from Trichosanthes kirilowii and T. laceribractea in Caenorhabditis elegans: A Comparative Analysis and Mechanism Study
Source: Antioxidants (Basel). 2024 Jul 18;13(7):861. doi: 10.3390/antiox13070861 (PMC11273834; doi:10.3390/antiox13070861)

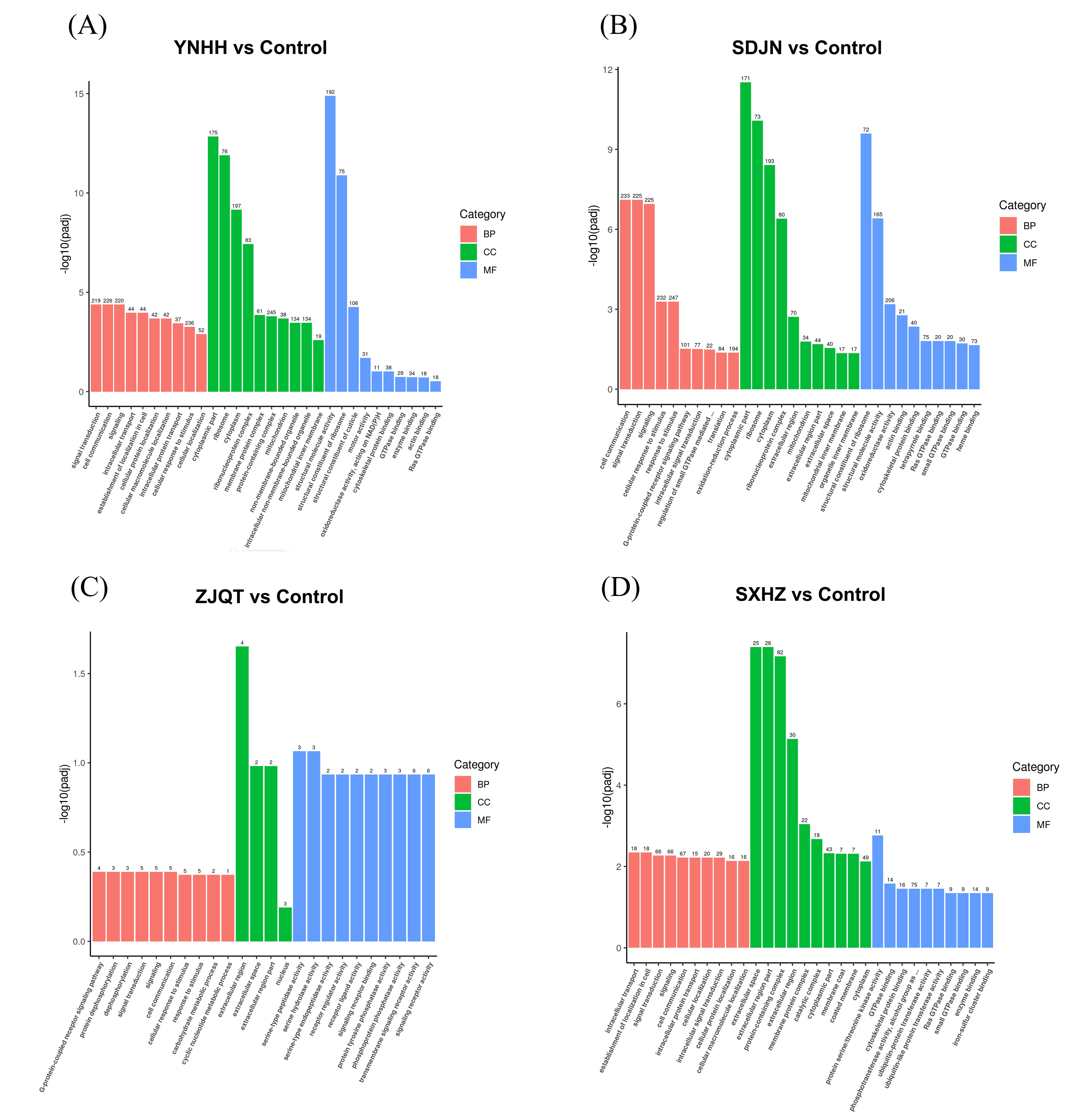

Supplement: Supplementary file 1 [file antioxidants-13-00861-s001.zip › Figure S1.TIFF]

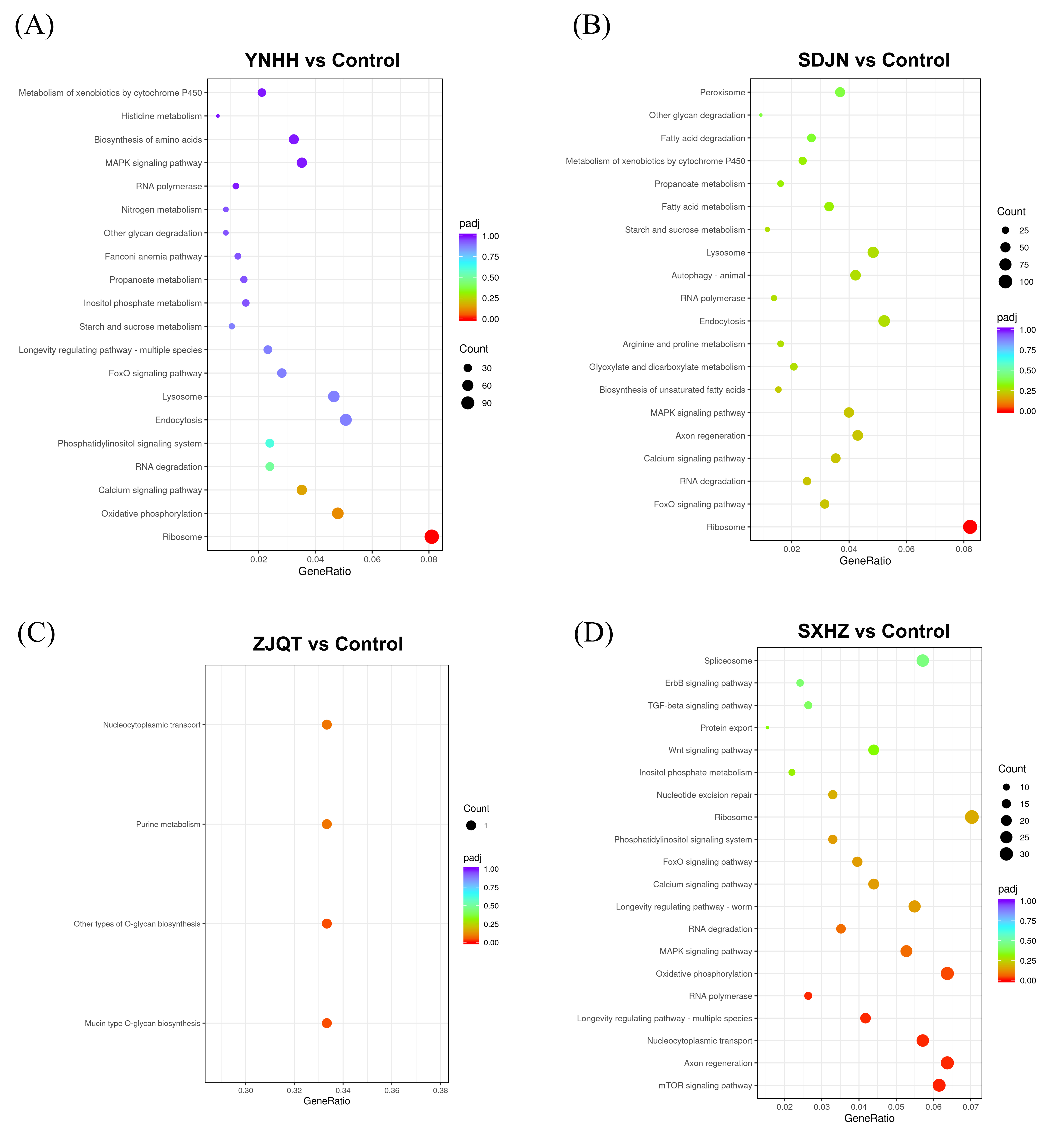

Supplement: Supplementary file 1 [file antioxidants-13-00861-s001.zip › Figure S2.TIFF]
